# Transparency: A central principle underpinning trustworthy guidelines

**DOI:** 10.1016/j.jclinepi.2021.11.025

**Published:** 2022-02

**Authors:** Nathan Ford, Rebekah Thomas, John Grove

**Affiliations:** Science Division, World Health Organization, Ave Appia 21, 1211, Geneva, Switzerland

## Abstract

• The development of trustworthy guidelines follows a range of principles.
• In response to the Covid pandemic there was an urgent need for guidelines to respond to a wide range of clinical and public health challenges; timelines became contracted and evidence was frequently lacking or of poor quality.
• For guideline development the World Health Organization, it became clear that one of the most important principles to adhere to is transparency as this underpins all other principles of guideline development.

Over the last 18 months the World Health Organization (WHO) has published over 300 guidelines across a range of clinical, environmental and public health areas to help decision makers around the world respond to the COVID19 pandemic [1]. WHO plays a central role by developing normative guidelines which provide a reliable starting point for the adoption or adaptation by national guidelines, particularly when time and resources are limited.

In 2007, WHO established a Guideline Review Committee in response to criticism that WHO guidelines were not based on the best available evidence and relied on expert opinion [2]. An evaluation of guidelines published after the establishment of the Guidelines Review Committee concluded that guideline development has become more systematic and transparent and that, to the extent possible, recommendations are based on evidence [3].

Guideline development at WHO follows internationally recognized methods and standards to which all approved guidelines must adhere [4]. These standards aim to ensure that guideline development is supported by an external group with gender balance and diverse expertise and geographic perspectives, that financial or intellectual conflicts of interests are identified and appropriately managed, and that the available evidence and any other relevant implementation considerations are systematically appraised to inform recommendations.

During the COVID19 pandemic these standards have been challenged by the need to rapidly identify and appraise the evidence with frequent updating required across a range of questions in order to formulate recommendations.

To have maximum uptake and impact, guidelines need to be trustworthy [5]. This is of the utmost importance during a public health emergency of international concern when governments around the world need guidance to direct action, and even more crucial when faced with a plethora of meaningful information, misinformation, and disinformation [6].

A central principle that underpins the trustworthiness of any guidelines is transparency. An emphasis on transparency ensures that each of the core steps is publicly available and can be adapted to different settings, but also challenged and ultimately improved. These steps include: what questions of uncertainty are being examined, what evidence was retrieved, who assessed this evidence – their expertise and any conflicts of interest that may exist – the balance of benefits, harms and other judgments for or against recommending a specific intervention, and who peer reviewed the draft guidance. Transparency underpins tools like the RIGHT Statement, a reporting checklist aimed at encouraging transparency to help end users understand the key steps that were followed when developing a guideline [7].

Guideline development starts by identifying important questions of uncertainty and implementing a structured appraisal process. The scope of a given guideline, including the formulating of key questions, is set by the technical leaders within WHO. In order to answer the key questions, relevant evidence is systematically appraised by independent researchers and the certainty of the overall body of evidence is assessed using tools such as Grading of Recommendations Assessment, Development, and Evaluation (GRADE) which assesses eight domains: five domains – risk of bias, reporting bias, imprecision, indirectness, and inconsistency – are used to rate down the certainty of the evidence, while the last three – large effects, plausible opposing residual confounding, and dose-effect relations – can be used to increase certainty [8]. An expert guideline development group, independent of WHO and supported by a guideline methods expert, reviews this evidence and formulates recommendations. Transparency in the process of moving from evidence to recommendations is further supported by the use of GRADE Evidence-to-Decision frameworks, which summarize benefits and harms and include explicit considerations about resource use, equity, acceptability and feasibility [9]. An external review group provides a peer review of the draft guidance prior to finalization.

Guideline development groups are often required to make decisions when evidence is not optimal. This has been a feature of guideline development during the COVID-19 pandemic, and normative bodies need to prepare for any emergent, unconventional future health challenge. Guidelines need to be developed rapidly in response to an emerging disease where there is sparse data, low-quality studies, weak study designs, and an exponential growth in the number of publications, including pre-peer review publications [[10], [11]–12]. Disagreements about how to interpret the evidence and what actions to take are expected, and so it is particularly critical to be transparent about what evidence is used and what processes are followed in order to formulate recommendations. The explosive growth in the scientific literature requires continuous reassessment that may impact the type of evidence used and can result in changes in policy. Transparency is critical for helping people to understand the rationale for such changes.

In the early months of the pandemic response, WHO was criticized for taking too narrow a view of the evidence (modes of transmission), insufficient representation of civil society on guideline panels (community mask wearing), and the lack of broader expertise and perspectives (ventilation and aerobiology). WHO has responded to these concerns by expanding the scope of the evidence assessed and including additional expertise within guideline development groups and peer review groups.

When a rapid response to a novel emerging public health concern is needed, it may be a necessary and acceptable trade-off that guideline processes are compressed. This may be understandable given the urgency of responding and the rapidly changing nature of the situation, and reinforces the importance of transparency so that end users are aware of the trade-offs that have been made. Flexible approaches are needed so that recommendations can be updated as new evidence accumulates, including by adopting living approaches to evidence appraisal and guideline development [13].

In a period of great uncertainty, when guidance is urgently needed and the evidence is limited and/or in constant evolution, transparency is of upmost importance. If key questions are not addressed, if the evidence assessment is too restrictive, incomplete, or lacking in key areas, if an expert group lacks specific expertise or a particular member has a perceived conflict of interest, end-users of the guideline should be made aware of such limitations to facilitate local decision-making. The recent interrogation of WHOs guideline development process underscores the importance of transparency in ensuring guidance is relevant, credible, and trustworthy, and can be easily adapted and implemented to different settings globally. A valuable area for future research would be to appraise guidelines developed during a period of acute emergency against quality standards [14] to understand what trade offs were commonly made, and which could be minimized, to inform future emergency guideline development processes.

WHO remains committed to transparency, and welcomes critical feedback for improving its guideline development and content.

## Author statement

All authors contributed to the drafting and revision of this manuscript

## Financial disclosure

This article was supported by general funds from the Bill & Melinda Gates Foundation

## Disclaimer

Views expressed in this article are those of the authors and do not necessarily represent the views of the World Health Organization.
